# How to generate graded spinal cord injuries in swine – tools and procedures

**DOI:** 10.1242/dmm.049053

**Published:** 2021-08-31

**Authors:** Mark Züchner, Manuel J. Escalona, Lena Hammerlund Teige, Evangelos Balafas, Lili Zhang, Nikolaos Kostomitsopoulos, Jean-Luc Boulland

**Affiliations:** 1 Department of Neurosurgery, Oslo University Hospital, Rikshospitalet, 0372 Oslo, Norway; 2 Department for Immunology, Oslo University Hospital, Rikshospitalet, 0372 Oslo, Norway; 3 Center of Clinical Experimental Surgery and Translational Research, Biomedical Research Foundation of Academy of Athens, 11527 Athens, Greece; 4 Institute for Experimental Medical Research, Oslo University Hospital and University of Oslo, 0450 Oslo, Norway

**Keywords:** Spinal cord injury, Large-animal model, Farm pig, Aachen minipigs, Surgery

## Abstract

Spinal cord injury (SCI) is a medically, psychologically and socially disabling condition. A large body of our knowledge on the basic mechanisms of SCI has been gathered in rodents. For preclinical validation of promising therapies, the use of animal models that are closer to humans has several advantages. This has promoted the more-intensive development of large-animal models for SCI during the past decade. We recently developed a multimodal SCI apparatus for large animals that generated biomechanically reproducible impacts *in vivo*. It is composed of a spring-load impactor and support systems for the spinal cord and the vertebral column. We now present the functional outcome of farm pigs and minipigs injured with different lesion strengths. There was a correlation between the biomechanical characteristics of the impact, the functional outcome and the tissue damage observed several weeks after injury. We also provide a detailed description of the procedure to generate such a SCI in both farm pigs and minipigs, in the hope to ease the adoption of the swine model by other research groups.

## Abstract (precis)

**Summary:** We present tools and procedures to generate a swine model for spinal cord injury, aiming for a correlation between injury severity, functional outcome and tissue damage.

## INTRODUCTION

Despite many decades of intensive research, a cure for spinal cord injury (SCI) remains elusive. Great advances in our understanding of the pathophysiology and the cellular mechanisms that impede recovery were gathered by experimentations on different animal models, including mouse, rat, dog, cat, rabbit, pig and non-human primates. However, the majority of studies were conducted on rats and mice (Sharif-Alhoseini et al., 2017). Rodents present many advantages, such as ease of genetic manipulation, ease of handling and quick recovery from invasive surgery. They also present substantial differences from humans, such as a tendency to recover spontaneously and quickly after SCI. This perturbs the evaluation of a treatment. These differences may account for the difficulties in translating promising results from basic research into an efficient clinical therapy (Tator, 2006). Hence, it seems necessary to take an additional step with an animal model closer to humans (Courtine et al., 2007; Kwon et al., 2015; Nardone et al., 2017; Schomberg et al., 2017). Although it remains to be proven, experimental treatment shown to be efficient in such a model might have a higher likelihood of being successful in clinical trials. There is an interesting trend to work with non-human primates (Beaud et al., 2012; Corre et al., 2017; Courtine et al., 2007; Darian-Smith et al., 2014; Ma et al., 2016; Nout et al., 2012a,b; Pritchard et al., 2010; Rosenzweig et al., 2010, 2018; Salegio et al., 2016). The advantages of non-human primate animal models is that they are similar to humans regarding the organization of the central nervous system, level of dexterity and possibility of bipedal locomotion. However, potential translational issues may arise from the small sizes of the species used [e.g. 2.5–15 kg Rhesus monkeys (Beaud et al., 2012; Darian-Smith et al., 2014; Friedli et al., 2015; Ma et al., 2016; Salegio et al., 2016), 2–7 kg African green monkeys (Pritchard et al., 2010; Slotkin et al., 2017), 5 kg cynomolgus monkeys (Ye et al., 2018), 0.7–1 kg squirrel monkeys (Bowes et al., 2012; Liao et al., 2018; Wang et al., 2016) and small mouse lemurs weighing 30–120 g (Corre et al., 2017; Poulen and Perrin, 2018)]. For example, the regrowth of axons over a few millimetres may have a potent effect in small animals, whereas it may only have a marginal effect in larger animals or in humans. Although pigs are not as close to humans as primates in some aspects (e.g. dexterity and locomotion), the similarities in anatomy of the vertebral column and spinal cord represent a good compromise (Lee et al., 2013; Schomberg et al., 2017). This explains the increased number of pig studies over the past decades (Benavides et al., 2017; Bernards and Akers, 2006; Hachmann et al., 2013; Hong et al., 2016; Huang et al., 2013; Jones et al., 2012, 2013; Kowalski et al., 2016; Kuluz et al., 2010; Kwon et al., 2015; Lee et al., 2013; Martirosyan et al., 2015; Navarro et al., 2012; Okon et al., 2013; Santamaria et al., 2018; Streijger et al., 2015, 2016, 2017; Tigchelaar et al., 2017; Zahra et al., 2010; Zurita et al., 2008, 2012). Despite this growing interest in the pig model, utilizing it requires a specific infrastructure, a substantial budget and several types of expertise that can be challenging to gather. Moreover, because incomplete SCIs in humans are associated with a better recovery prognosis, it is of capital importance that such incomplete spinal lesions are consistently reproduced in the pig model.

In a previous work, we reported the development of a new impacting apparatus that is composed of a spring-load impactor, a spinal cord support system and a vertebral column suspension system attached to a pig operating table (Züchner et al., 2019). Because human traumatic SCI often originates from an initial contusion followed by prolonged compression, we designed an impactor that could generate an initial impact followed by static compression. To promote the adoption of this model by other research groups, we felt that a complete methodological description would be a useful resource. In this paper, we present a detailed protocol for engineering the impactor complemented by a computer-aided design (CAD) file for replication and a dedicated MATLAB code with a graphical user interface for intra-operative analysis of impact characteristics. We also provide a complete description of the surgical procedure to create a contusion SCI and the post-operative care of farm pigs and Aachen minipigs. We also present a method for the evaluation of the functional outcome based on an already established scoring system combined with objective measurements. Our approach was to test different injury strengths until we obtained a functional outcome corresponding to incomplete sensory and motor paralysis, mimicking human incomplete SCI.

## RESULTS

### Impact characteristics and locomotor behaviour

**Fig. 1.**
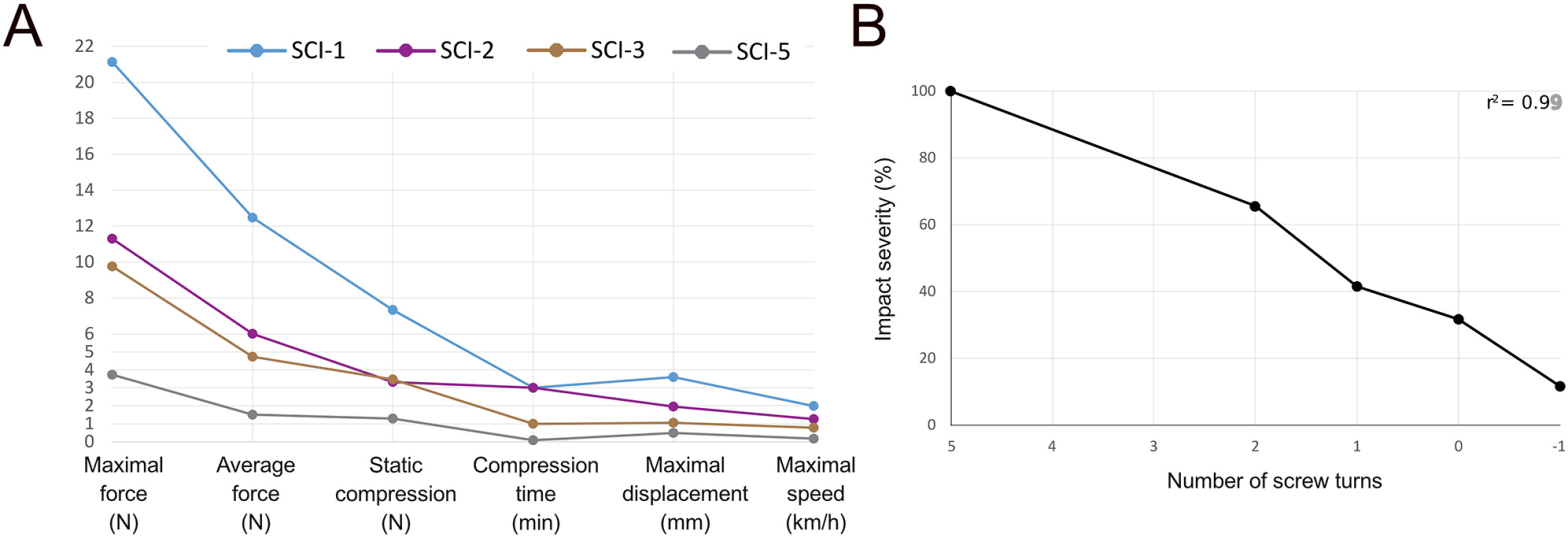
**Impact severity.** (A) Different impact parameters are used to characterize the severity of impact. After normalization of the parameters and averaging of interdependent parameters, a grand average returns a single value that qualifies the impact severity. (B) Impact severity versus the number of turns of the tension adjustment screw.

**Fig. 2.**
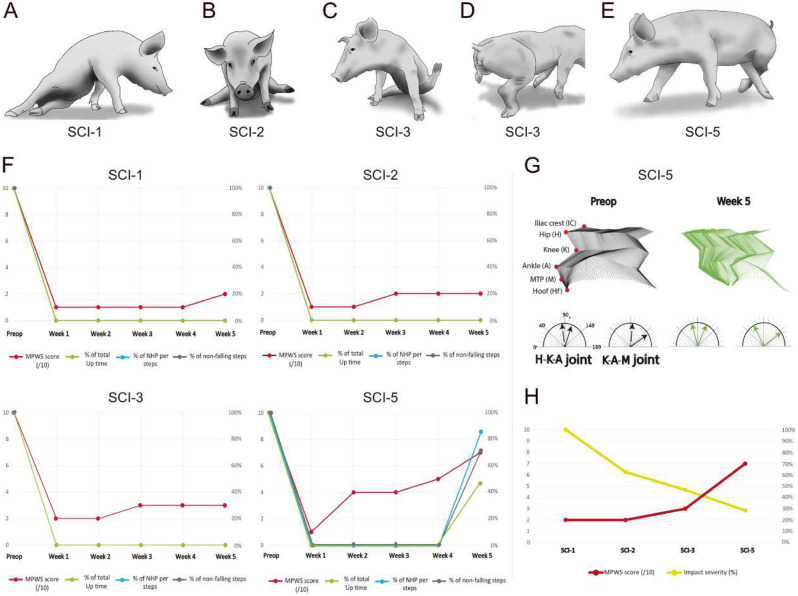
**Evaluation of locomotor outcome.** (A) Paralyzed pig with no visible movements and no trunk control. (B) Low-amplitude hindlimb movement. (C) High-amplitude hindlimb movement. (D) Standing with improper hoof placement. The dorsal part of the hoof is pushed against the ground instead of the plantar part. (E) Improper stepping with improper hoof placement as in D. Such a step often results in a fall. (F) Weekly evaluation of the locomotor behaviour for all farm pigs using the Miami porcine walking score (MPWS), the percentage of time standing, the percentage of normal hoof placement (NHP) and the percentage of non-falling steps. Note that only SCI-5 recovered limited stepping abilities 5 weeks after the lesion. (G) Kinematic and joint-angle analysis of a step performed by SCI-5 during the evaluation at week 5. The arrows represent the minimal and maximal angles. (H) Impact severity and MPWS at 5 weeks have an inversely proportional relationship.

We aimed to generate an incomplete swine model for SCI to test new experimental treatments. A first necessity was to identify the appropriate injury severity that results in an incomplete SCI. A goal of this research was to obtain a pig capable of stepping, although with deficits still present, at 5 weeks post-injury. We started with five farm pigs, referred to as SCI-1 to SCI-5. Our previous study showed that an impact of 46 N with our spring-loaded impactor was creating such serious tissue damage that it was likely to be beyond any repair (Züchner et al., 2019). Therefore, as a first trial, we decided to reduce the strength by half. For SCI-1, we set the tension adjustment screw to five turns and obtained 21.1 N maximum force, 12.5 N average force, 7.3 N static compression for 3 min, 3.6 mm displacement, 2 km/h impact speed and 14.4 ms impact duration (Fig. 1). The outcome of this injury strength over a 5-week recovery period was complete paralysis with little to no signs of functional improvement. The main feature was paraplegia with no trunk control below the injury [i.e. Miami porcine walking scale (MPWS) score≤2 points], resulting in the rump positioned on the floor sideways (Fig. 2A,F). Urinary retention was observed immediately after injury, and a transurethral catheter had to be used for a week, after which the bladder became reflexive. As this injury was too strong, we next reduced the tension in the spring down to two screw turns, and obtained a reduction in all impact parameters so that the impact severity was down to 65.6% from SCI-1 (Fig. 1B). Immediately after the injury, the functional outcome was similar to the previous pig. However, within a few days, SCI-2 regained control of its lower trunk muscles, and it could get itself into a ‘sitting’ position. Within 2 weeks, SCI-2 responded to skin pinches on the hindlimb, and it was capable of small amplitude hip joint flexion to raise the limb against gravity ∼5–15 cm above the ground for 2–10 s (Fig. 2B). However, the other limb joints were locked in an extended position. As for SCI-1, it was necessary to place a transurethral catheter for the first week. To further generate a lighter injury, we adjusted the screw down to one turn and shortened the duration of the static compression. This reduced the impact severity down to 41.5% from that of SCI-1 (Fig. 1). SCI-3 rapidly recovered lower trunk control and could easily get into a sitting position. Rapidly, SCI-3 was able to flex the hip and knee joints, resulting in movements of a certain amplitude (Fig. 2C). The pig also made attempts to stand and was able to withstand gravity for less than 2 s when manually raised to a standing position (Fig. 2D). Like SCI-2, it also responded to skin pinches below the injury. In contrast to SCI-1 and SCI-2, there was no need for a urinary catheter. Although the locomotor outcome of SCI-3 was mimicking motor incomplete human patients, the function was too impaired with respect to our initial aim. To obtain an overview of the severity scale, we generated two new lighter injuries, SCI-4 (0 screw turn) and SCI-5 (−1 screw turn). This −1 value results from a screw turn in the opposite direction (i.e. counterclockwise) from the established 0 value, further reducing the tension of the impactor spring. As expected, the impact severities were decreased to 31.7% for SCI-4 and 11.6% for SCI-5 (Fig. 1B). Because SCI-4 had a lighter impact severity than SCI-3, we did not anticipate the need for a urinary catheter. However, SCI-4 died during the early post-operative period, and the autopsy revealed a bladder rupture. For this reason, there are no locomotor behaviour data for this animal. The day following the injury, SCI-5 was able to rise and stand unsupported and could make one to two steps before falling down. However, the situation rapidly degraded during the next few days. One week after the injury, the pig was only able to make small-amplitude movements, just as SCI-2 (Fig. 2B,F). A possible reason for this deterioration is discussed later in light of MRI evidence. By the second week after injury, SCI-5 scored up to four points on the MPWS, and it was able to perform large amplitude movements. At week 4, SCI-5 was able to withstand gravity for several seconds and to step while leaning against the wall. At week 5, SCI-5 was partially ambulant and able to stand half of the time during the evaluation (MPWS=7 points), although with improper limb and hoof positioning (Fig. 2E,F). We further assessed stepping at week 5 compared to pre-operative recordings by tracking limb joints (Fig. 2G). Kinematic analysis showed that the flexion of the hip joint was increased during the entire step. Taken together, these data showed that impact severity has a linear relationship with the number of screw turns (*r*^2^=0.99) (Fig. 1B), and that it produces a graded functional outcome that can be used to mimic incomplete SCI (Fig. 2H).

### Tissue damage

**Fig. 3.**
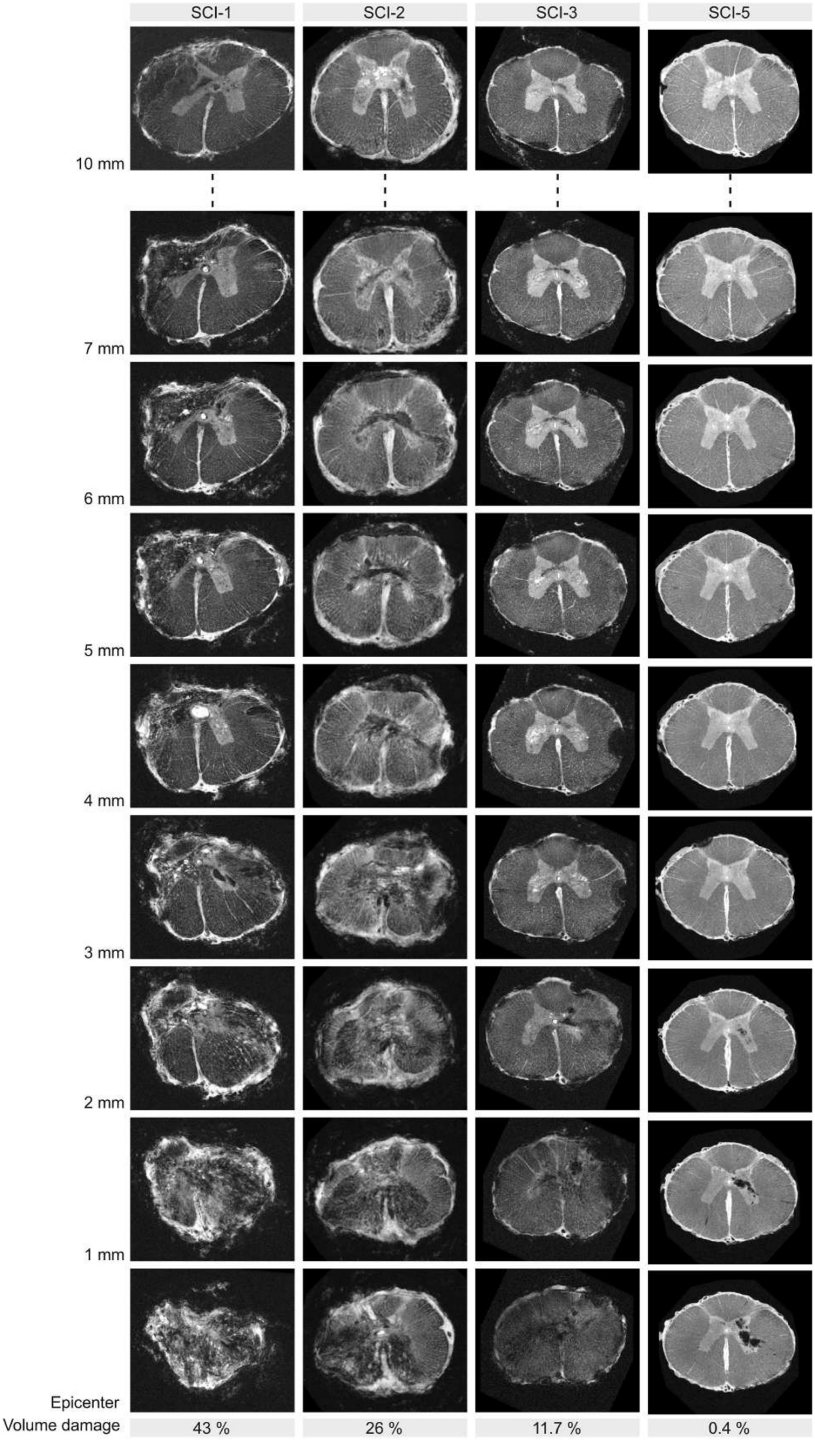
**High-resolution MRI scans for SCI-1, SCI-2, SCI-3 and SCI-5 from 0 to 10 mm distance from the injury epicentre.** Quantification of volume damage correlates with impact severity.

After dissection, the spinal cords were prepared for 9.4T MRI and scanned (Fig. 3). T1-weighted images for SCI-1 showed profound tissue damage affecting all parts of the spinal cord at the injury epicentre. Signs of lesions were visible over an extensive rostro-caudal part of the spinal cord covering ∼10 mm in each direction. About 43% of the spinal volume showed signs of damage (Fig. 3). Less-severe tissue damage over a smaller rostro-caudal distance was found for SCI-2, with 26% damage. In the same line, the MRI for SCI-3 showed yet milder and more-restricted tissue damage, with 11.7% volume damage. Hence, there is a clear correlation between the impact severity and the lesion severity. Interestingly, SCI-5 presented just a little tissue damage (0.4%), which was mostly restricted to the grey matter of the spinal cord. This dense signal drop is characteristic of bleeding and potentially post-operative re-bleeding, which could explain the worsening of this pig during the first post-operative week.

**Fig. 4.**
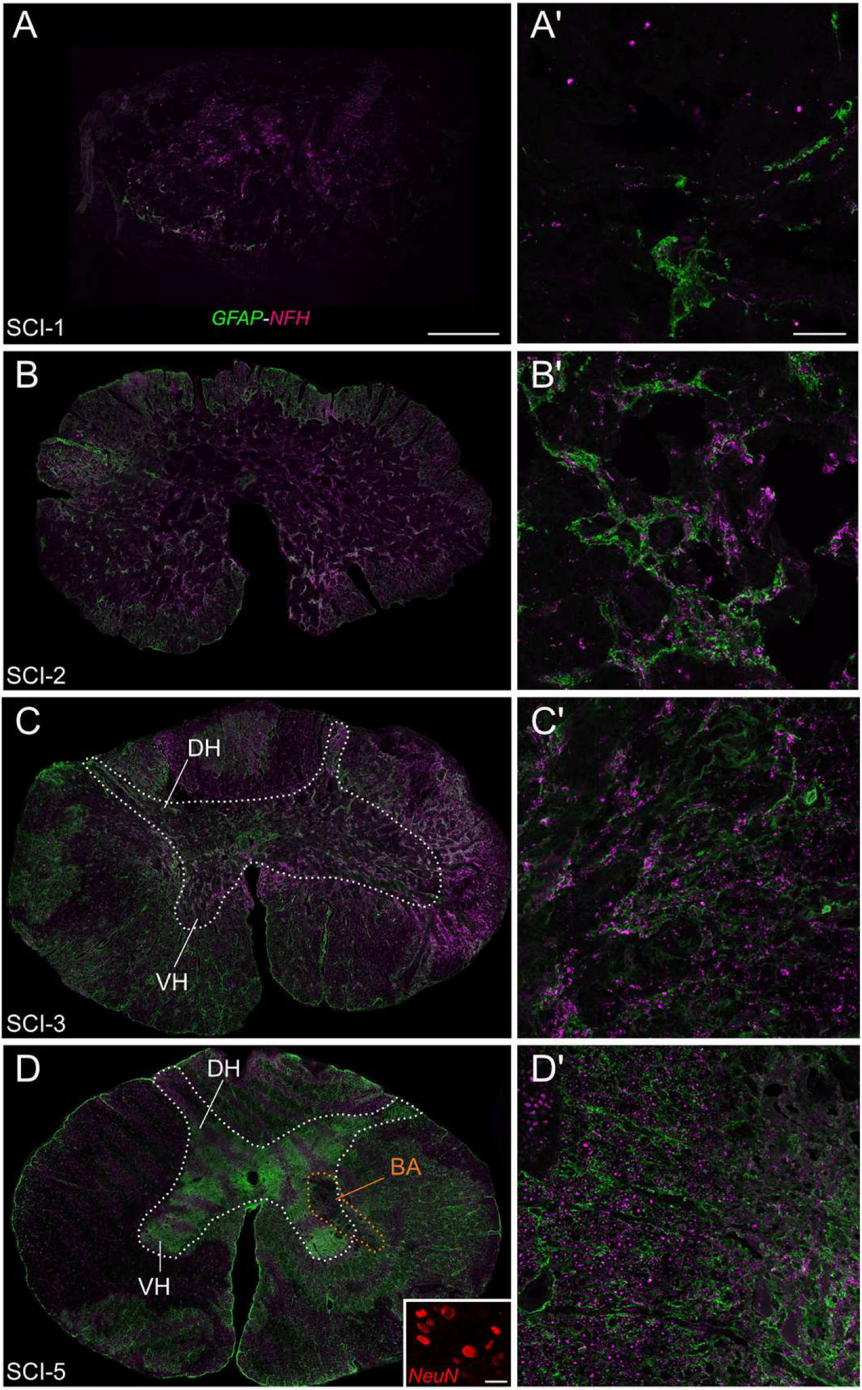
**Tissue damage revealed by immunofluorescence.** (A-D′) Immunofluorescence for GFAP (green), NFH (magenta) and NeuN (red) from the lesion area for SCI-1 (A,A′), SCI-2 (B,B′), SCI-3 (C,C′) and SCI-5 (D,D′). The white dotted line in C outlines the presumptive grey matter. The white dotted line in D outlines the grey matter and the orange dotted line outlines the bleeding area, also observed with MRI (Fig. 3). The inset in D shows NeuN-positive cells of the dorsal horn. BA, bleeding area; DH, dorsal horn; VH, ventral horn. Scale bars: 1 mm (A), 100 µm (A'), 25 µm (inset in D).

Although high-resolution MRI reveals important information about the extent of tissue damage, it does not reflect changes within different cell populations. Therefore, we labelled spinal cord sections with antibodies against glial fibrillary acidic protein (GFAP) for astrocytes, neuronal nuclear antigen (NeuN) for neurons, and neurofilament high (NFH) for nerve fibres. Consistent with the MRI scans, there was extensive tissue damage at the lesion site of SCI-1. Different regions of the spinal cord could not be identified with certainty (Fig. 4A). The surface of the tissue was 14.3 mm^2^. Although intensely labelled, only a few cells were positive for GFAP. No NeuN-positive cells were visible. Although individual NFH-positive nerve fibres were visible, NFH labelling was also disorganized, sometimes forming aggregates (Fig. 4A′). The lesion area of SCI-2 was less severely damaged than that of SCI-1 (Fig. 4B). The outline of the spinal cord was clearer, with a surface area of 20.3 mm^2^. The delimitation between the white and grey matters was visible in most parts of the section but no other spinal structures could be identified. The immunolabelling for GFAP was mostly found in the white matter. The staining was dense, resembling reactive astrocytes (Fig. 4B′). No NeuN-positive neurons were visible. The staining for NFH was inhomogeneously distributed, but globally denser than for SCI-1 and with more individual nerve fibres. The lesion area of SCI-3 was less severely damaged than that of SCI-1 and SCI-2 (Fig. 4C). The outline of the spinal cord was clearly visible, and the surface area of the tissue was 26.1 mm^2^. The delimitation between the white and grey matters was visible in many parts of the spinal cord. However, consistent with the MRI scans, a clear shape of the grey matter was not visible on either side of the spinal cord. In contrast with SCI-1 and SCI-2, the staining for GFAP was more homogeneously distributed, including in the presumed areas of the grey matter. In several areas, the staining consisted of thinner, less densely packed astrocytic-like processes, suggesting reduced astrogliosis (Fig. 4C′). No NeuN-labelled neurons were visible in the dorsal and ventral parts of the spinal cord. Although still inhomogeneously distributed, individual nerve fibres were present in all parts of the spinal cord. However, intense aggregates of NFH were still visible. Consistent with the MRI scans, the lesion area of SCI-5 was the least damaged of this series, with a clear outline, clear boundaries between the white and grey matters, and clear shapes of the dorsal and ventral horns and the central canal (Fig. 4D). The surface area of the section was similar to that of SCI-3, 25.7 mm^2^. The shadow of a bleeding on one side of the grey matter with the same shape as seen in the MRI scan was visible (orange dotted line outline in Fig. 4D). The staining for GFAP was more intense in the grey matter, and mostly thin individual cell processes were visible in the white matter (Fig. 4D′). NeuN-positive cells were visible in the grey matter especially in the dorsal horn (inset in Fig. 4D) and only a few NeuN-labelled cells were found in the ventral horn. The NFH staining was denser and more homogeneously distributed than in all other sections.

Altogether, the observations of the tissue with high-resolution MRI and with immunofluorescence provide complementary results showing different aspects of the damage. As expected, the severity of the lesion was consistent with the impact severity.

### Transitioning from a farm pig toward a minipig animal model

During this study, the weight gain of farm pigs, typically from 20 kg to 40 kg, became problematic for daily handling. This also limited the post-operative observation to 5–7 weeks. For SCI-3, X-rays showed that the rostral pedicle screw was displaced (Fig. S1A). Furthermore, during the dissection of the spinal cord (Fig. S2), we noticed that the rostral screw of SCI-3 was not firmly implanted anymore. Despite this, we did not detect any signs of instability. We presumed that the permanent sitting position combined with the weight gain produced too much load force on the implant, resulting in its failure. Therefore, for all remaining pigs, we kept the implants on both sides in place to better stabilize the vertebral column (Fig. S1B).

**Fig. 5.**
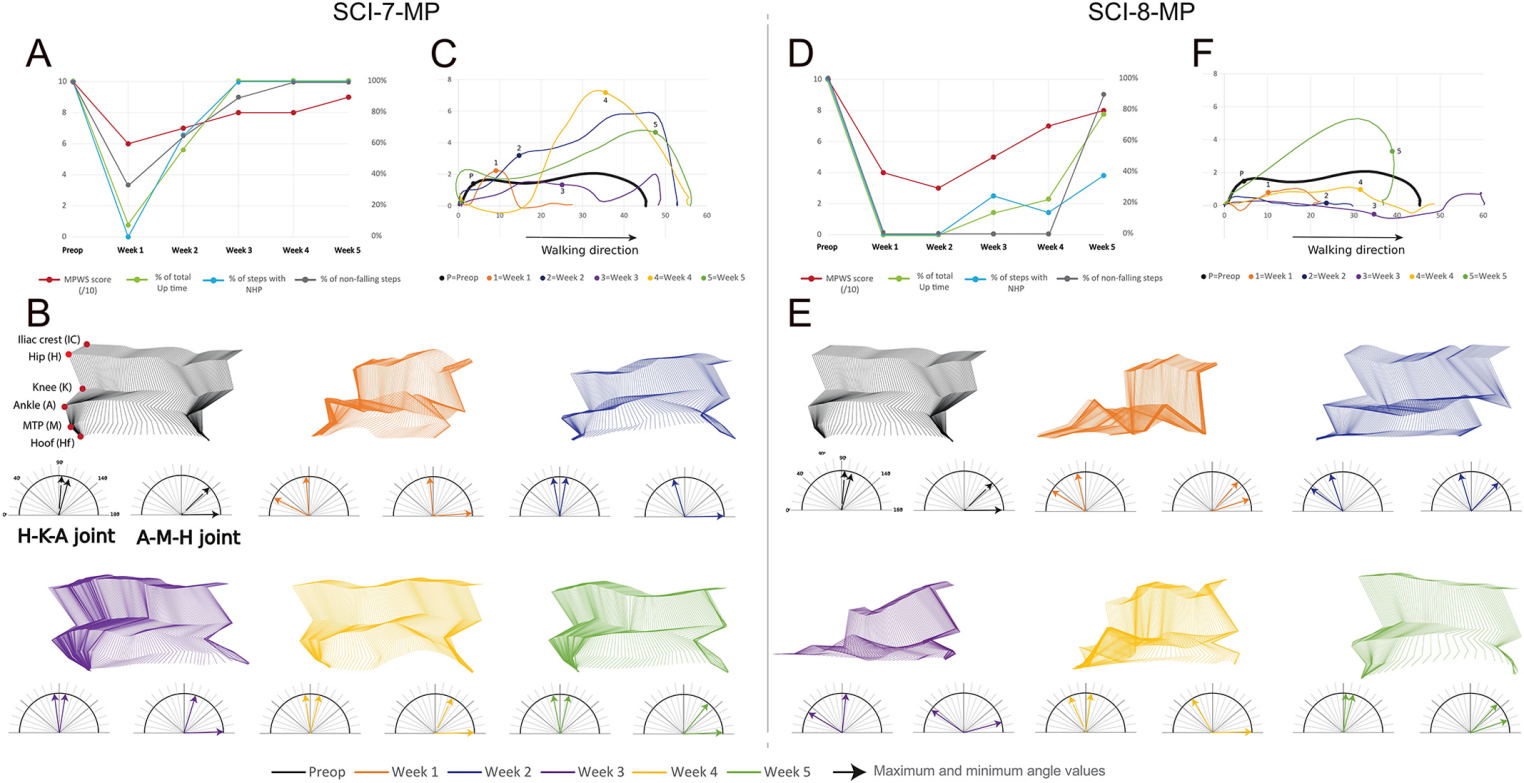
**Walking characteristics of two Aachen minipigs injured with a light and a medium impact.** (A,D) Weekly evaluation of the locomotor behaviour using the MPWS, the percentage of time standing, the percentage of NHP and the percentage of non-falling steps. (B,E) Stick diagrams representing the kinematic for the best step recorded. Each week is represented by a different colour. Maximum and minimum joint-angle values (arrows) are shown for each week. (C,F) Hoof trajectory tracked during the swing phase of a step cycle before and after the spinal lesion. MPWS, Miami porcine walking scale; NHP, normal hoof placement.

Because of these issues related to farm pigs and in order to ensure a more stable model of incomplete SCI, we decided to move the focus towards the Aachen minipigs model, using three animals (SCI-6-MP, SCI-7-MP and SCI-8-MP). We assumed that most of what we learned from farm pigs would be applicable to minipigs. We first performed an acute experiment in a minipig (SCI-6-MP) to test our apparatus and to address possible differences in anatomy or anaesthesia requirements. The most noticeable difference that we found was bone stiffness. Bone removal during laminectomy was more difficult, especially the puncture of the pedicles, which required an electrical drill. Next, we performed injuries of two different strengths, aiming for a light impact severity (22%) for SCI-7-MP and a medium impact severity (39%) for SCI-8-MP. The recovery profile over the 5-week period was different. For SCI-7-MP, at 1 week after injury, limb function was reduced, but not lost (Fig. 5A). There was rapid improvement during the following weeks, and, at week 5, limb function was close to normal. The kinematic analysis shows the progression of the recovery with a gradual trend towards normalization of the limb joint angle during the different stepping phases (Fig. 5B). Despite this finding, the analysis of the hoof trajectory shows that, even at week 5, there was an abnormal pull of the hoof upward (Fig. 5C). It is important to note that the kinematics and the trajectory were always analysed based on the best stepping recorded. By contrast, the recovery of SCI-8-MP was not as fast, and even at week 5, ∼10% of steps still resulted in a fall and there was ∼60% dorsal hoof placement (Fig. 5D). This was also evident from the kinematic analysis. It showed improper stepping, or rather crawling with inappropriate joint angle values that tended to improve at week 5 (Fig. 5E). The trajectory of the hoof had a small amplitude up to week 5, where it surpassed the control trajectory, especially in the upward direction (Fig. 5F).

## DISCUSSION

In this article, we show that it is possible to generate a model of incomplete SCI in both farm pigs and minipigs. We also present data that justify transitioning from farm pigs toward a minipig model. The methodology and the construction of a spring-load impactor and the surgical and post-operative procedures to generate a controlled and graded SCI are presented in detail.

### Impact severity

**Fig. 6.**
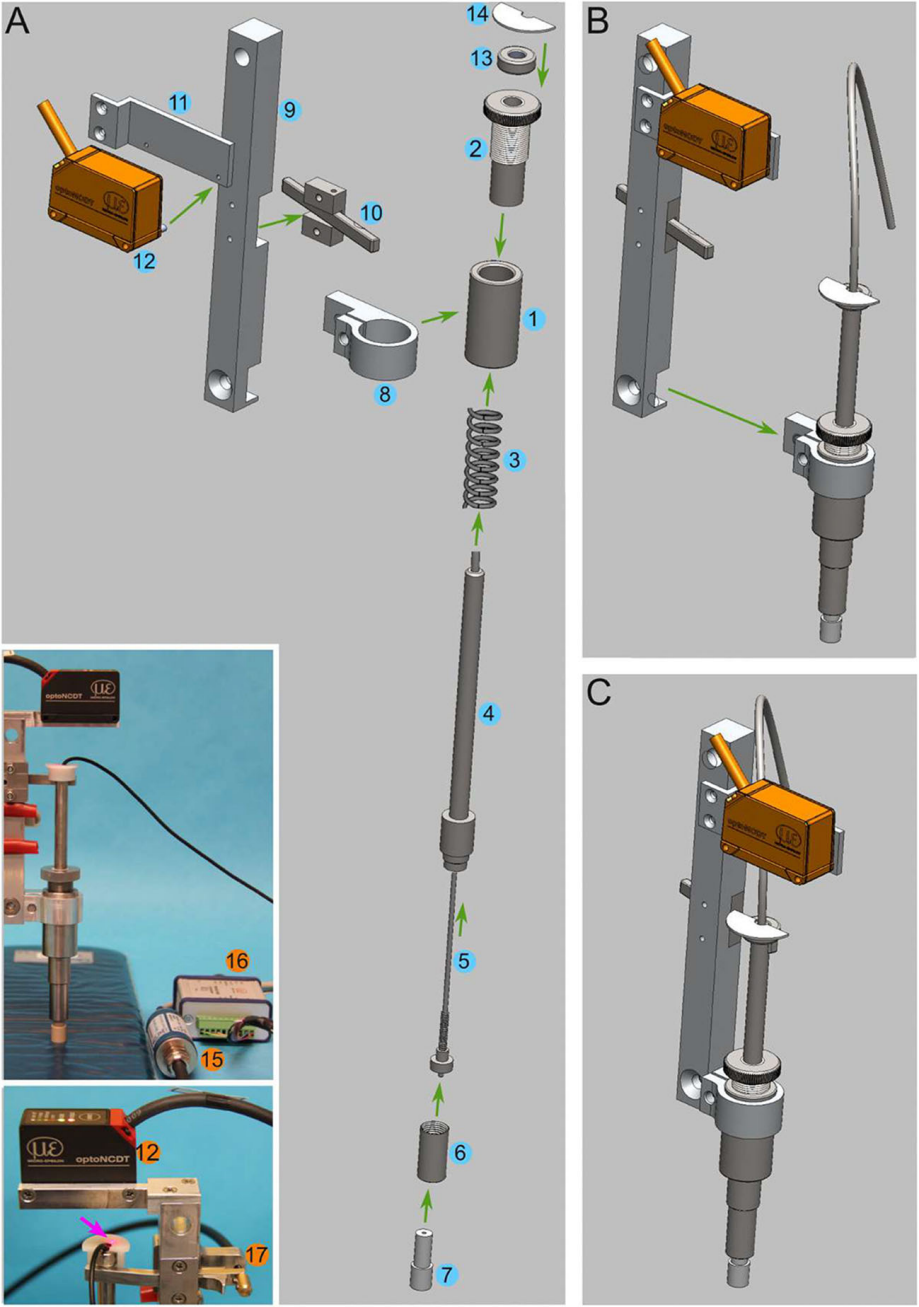
**A step-by-step assembly of the impactor.** (A) Components of the impactor: (1) Main sleeve; (2) tension adjustment screw; (3) compression spring; (4) plunger; (5) load cell sensor; (6) nut cylinder; (7) impactor tip; (8) cylinder clamp; (9) bar; (10) release mechanism; (11) laser sensor platform; (12) laser displacement sensor; (13) nut; (14) white plastic semi-disc; (15) load cell amplifier; (16) laser sensor control unit; (17) bicycle wire attached to the release mechanism. (B) Intermediate steps in assembly. (C) Assembled impactor. The complete assembly procedure is described in the Materials and Methods section.

In a previous study, we described the development of an impacting apparatus to generate SCIs in large animals (Züchner et al., 2019). We demonstrated that our spring-load impactor generates graded and reproducible impacts on gel pads. We further demonstrated, in acute pig experiments, that our complete apparatus generated biomechanically reproducible injuries. Post-mortem analysis of the spinal cord tissue also showed consistent tissue damage. In the present article, we further detail the engineering of the impactor with a construction figure (Fig. 6) and a CAD file (Dataset 1). This should allow for easy reproduction of the impactor by any workshop. In addition, we also provided a MATLAB code with a graphical user interface to perform the analysis of the output of the sensors during the surgery (Dataset 2). This opens the possibility of making a decision to terminate the experiment in case of an inaccurate injury. The program performs calculations for the main impact peaks and the secondary peaks (rebounds). This includes the maximal and mean force, the maximal instant force increase and decrease, the duration, the impulse, the static compression force, the total displacement, the compression displacement and the speed.

Although these provide a complete characterization of the impact, it does not reflect the global picture. Consequently, it is tempting to compare different impacts on the basis of a single parameter (e.g. the maximal impact force). However, there is no obvious reason to prefer one parameter over another. Accordingly, there was a need for data reduction to obtain a single value to characterize the impact. The values from these different parameters vary greatly, and they are expressed in different units. Therefore, we normalized them against reference values, and expressed them as a percentage. Because SCI-1 was paralyzed and showed almost no improvement with time, it seemed appropriate to use it as a reference, especially because our aim was to generate an incomplete SCI. It was tempting to average all the parameters, but, although indicative, the average force, the impulse, the impact duration and the displacement are dependent on a subjective user's choice for the start and end position of the impact peak. To obtain the most reliable calculation, we have eliminated them and averaged the remaining factors that do not depend on user input. These are the maximum force, the static compression force, the maximum speed and the compression time. The average of these parameters resulted in a single value that we called the impact severity, expressed as a percentage of SCI-1. This approach allowed us to characterize the impact based on multiple parameters, with the convenience of a single value for the comparison of different impacts.

### Key steps for the surgical procedure

To date, several groups have developed pig models for SCI using contusion, transection, compression, spinal shortening and spinal distraction (Bernards and Akers, 2006; Hachmann et al., 2013; Kuluz et al., 2010; Lee et al., 2013; Lim et al., 2010; Modi et al., 2011; Navarro et al., 2012; Santamaria et al., 2018; Skinner and Transfeldt, 2009; Solis et al., 2013; Zahra et al., 2010; Zurita et al., 2012). However, when we decided to use a contusion pig model to test experimental treatments, we found that the different descriptions available in the literature were not sufficient to replicate a model in our facility. Thus, one aim of this article is to provide a complete description of our impacting apparatus and a step-by-step surgical protocol so that other groups can duplicate and eventually improve these methods. Admittedly, the surgery is relatively complex and it requires a team of experts. In our case, we operate with a veterinary anaesthetist, a neurosurgeon and an assistant or veterinary nurse. In addition, if intra-operative neuromonitoring is required, it is an advantage to have a neurophysiologist. Although with some training, the anaesthetist may also perform the recordings in parallel with the monitoring of vital functions. A trained neurosurgeon or an orthopaedic surgeon will have no difficulties up to the laminectomy. The critical part of this procedure is the positioning of the spinal cord support system. This requires a relatively large laminectomy with partial removal of the facet joint. The anatomy of the pedicles in farm pigs or minipigs is not exactly identical to that of humans, and it requires some training to precisely implant the pedicle screws. To puncture the pedicle, the small pedicle probe (Fig. S3C, element 12) was sufficient in farm pigs. However, it was almost impossible to puncture the pedicle of minipigs without an electrical drill. In general, we found minipig bones to be denser than those of farm pigs of the same weight. This is likely due to bone maturation that is not completed in 20 kg farm pigs. However, our experience is limited to Aachen minipigs, and we do not know whether this is also valid for other breeds of minipigs. A precise positioning of the pedicle screws is important to avoid a conflict with the placement of the link element and the plates. It is important to plan the placement of the different elements to avoid a conflict with the nerve roots. It has never been necessary to cut a root in this process. We also recommend positioning the curved plate before the implantation of the last screw (T13 on the right side). This will help in deciding the appropriate angle and laterality for the placement of this screw and in avoiding conflict with the plate. In all cases, this step requires training; some of it could primarily be done on pig cadavers. Note, a cadaver not bled could spoil the training because of hardly controllable post-mortem bleeding.

### Transition from farm pigs to minipigs

During the 5 weeks of experimentation, the farm pigs doubled their weight, which increased the difficulties in the daily handling of the animals. This rapid weight gain also restricted the maximum duration of the experiment to ∼5–7 weeks, which may be too short for the evaluation of some chronic treatments. In addition, the weight gain may impede functional recovery, as the muscle mass reduction due to hypoactivity combined with the weight doubling may impede any attempt to stand. Moreover, this weight gain, together with the sitting position of the pig, could create a force that exceeds the tolerance of the implants or the bone in which they are positioned. The surgery involves partial removal of the facet joint, potentially creating instability in the vertebral column. We originally assumed that a unilateral fusion would provide sufficient stabilization, and, to leave as few implants as possible, we removed the implants on the left side. However, we observed one implant failure, indicating that the load was greater than we anticipated. Increasing the stabilization by leaving the implants bilaterally appeared to be efficient as we did not observe further implant failure, although we did not have enough observations to ascertain it. Altogether, these negative aspects clearly undermined the farm pig model for SCI. For these reasons, we decided to migrate the model towards minipigs, despite their greater purchasing cost. Their main advantage is a slow growth rate with a reasonable plateau effect. They can also eventually reach 45 kg or more, but, according to the provider, it should take 24 months or more (https://www.carfil.eu/en/product/aachen-mini-pig-0696). By contrast, farm pigs can reach more than 200 kg during the same period. This represents an advantage for experiments with a duration that exceeds 5 weeks. It should be noted that different breeds of minipigs also have different growth rates.

### Locomotor behavioural evaluation

The evaluation of the severity of the injury is crucial, as it is the most important functional output. Several groups have developed different solutions. However, they all rely on scoring motor function during a specific observation. Lee et al. (2013) developed the porcine thoracic injury behavioural scale. This scores the animal during hindlimb drag, stepping and walking on a 5 m long mat, filmed from behind as it walks away from the camera. Another group created a 14-point scoring system called the porcine neurological motor score (Navarro et al., 2012) that was designed to assess limb joints and the degree of ambulation. Forelimb–hindlimb coordination was further tested in animals with high scores (13–14) by the evaluation of stepping over a wooden bar. Other groups have developed other scoring systems based on open-field evaluations (Kuluz et al., 2010; Santamaria et al., 2018; Zurita et al., 2008). Therefore, there was no need to develop yet another scoring method for open-field assessments. We used the Miami swine motor scale in combination with other measurements; the percentage of standing time together with the percentage of non-falling steps provides a good evaluation of pig strength and balance. The percentage of normal hoof placement (plantar placement), as opposed to dorsal hoof placement also observed by other groups (Lee et al., 2013; Santamaria et al., 2018), provides a solid indication of hyperextension and quality of stepping. As all these parameters are presented as percentages, it is possible to average them and obtain a reduced value that is a convenient tool to assess global locomotor function. For ambulatory pigs, based on our previous work in mice (Züchner et al., 2018), we have developed a MATLAB analysis program for individual steps and angle variation. This allows further comparison of different animals or the longitudinal evolution of a specific animal. Such a complete analysis of locomotor function is a powerful tool for the assessment of spontaneous and treatment-induced recovery after SCI. Of note, a treadmill-based gait analysis method has recently been developed in healthy minipigs (Boakye et al., 2020), with the idea to further use it to analyse the outcome from SCI.

Altogether, the results presented in this article show that our spring-loaded impactor can generate graded SCIs in farm pigs with the impact severity matching both the functional outcome and the severity of the tissue damage as shown by MRI scans and immunolabellings. We also showed that the procedure used for farm pigs can easily be used for minipigs, and it is possible to generate a model of incomplete SCI in both breeds. We obtained a pig capable of stepping, although with clear deficits. This could constitute a possible model for therapies or rehabilitation research close to human patients with ASIA C or D, in which rehabilitation or new therapies found in other animal models could be tested more efficiently. Future studies will aim at demonstrating the long-term effects of the injury and to further characterize the cellular and molecular changes.

## MATERIALS AND METHODS

### Construction of the impactor

The development of a spring-load impactor has previously been described elsewhere (Züchner et al., 2019). We now provide a CAD file as Supplementary Information (Dataset 1). It was generated with Solidworks (Dassault Systemes), and it can be opened with a free viewer, eDrawings Viewer (Dassault Systemes; https://www.edrawingsviewer.com/download-edrawings). With this file, any mechanical workshop can duplicate the impactor. In addition, Fig. 6 provides details for the assembly of the impactor. It is constructed around a main sleeve (1) and the tension adjustment screw (2) (Fig. 6A; Movie 1). A compression spring (3; C04200452250S, Sodemann Industrifjedre A/S, Viby, Denmark) is positioned around the plunger (4). The plunger is further fit through the sleeve and the tension adjustment screw. The wire (5) of the load button sensor (LLB215, Futek, Irvine, CA, USA) is inserted through the plunger. Next, a nut cylinder (6) is attached to the plunger to secure the load cell sensor. This serves as a load button sensor house. The impactor tip (7), which we made in different diameters, is inserted through the sensor house and tightened onto the load button sensor. A cylinder clamp (8) is attached to the main sleeve on one side and bolted to a bar (9) on the other side (Fig. 6B,C). The bar serves as an arm onto which a release mechanism system is attached (10). Next, the laser sensor platform (11) and laser displacement sensor (12; ILD1420CL1, Micro-Epsilon, Ortenburg, Germany) are attached to the arm. A nut (13) is attached at the top of the plunger. This is used as a stopper for the lock/release mechanism. A white plastic semi-disc (14) is glued on top of the stopper nut. This is used as a reflective surface by the laser displacement sensor to triangulate the vertical position of the plunger. The wire from the load button sensor is plugged into the amplifier provided by the manufacturer (15) and connected to a computer via a USB port. It is controlled by the VS3 program (Lorenz Messtechnik GmbH, Alfdorf, Germany), which is also provided by the manufacturer. At the first start, the data sampling rate needs to be set to the maximum (5 kHz). This will be saved for subsequent sessions. The laser displacement sensor is connected to a controller (16) and plugged into the computer via a USB port. Again, it is important to configure the sampling rate to the maximum (4 kHz). This is done in the configurations of the optoNCDT 1420 software (Micro-Epsilon). This will also be saved for subsequent sessions.

### Analysis of impact characteristics

The measurements from each of the sensors can be output as .txt or .csv files and further analysed on different platforms. One possibility is to run the analyses intra-operatively to get the relevant information and eventually act on it (e.g. termination of the experiment). For this, we have written a MATLAB (MathWorks) code that analyses the sensor output files and returns the complete results in a few minutes. To make this experience user friendly, we also built a graphical user interface. We provide an executable file in the Supplementary Information (Dataset 2) along with a user instruction video (Movie 2), two demonstration files (Dataset 3) and a user manual (Dataset 4). After the analysis is completed, the program returns numerous biomechanical impact parameters. To reduce them to a single value, only core parameters composed of the maximum force, compression force, compression time and maximum speed were selected, because they can be obtained without user input. They were next normalized against the values of an impact that resulted in complete paralysis of the pig and averaged. This single value given as a percentage is the impact severity.

### Animals

All animal experiments described in this article were performed in the Surgical Unit of the Centre for Clinical, Experimental Surgery and Translational Research of Biomedical Research Foundation of the Academy of Athens, and were evaluated by the Project Evaluation Committee of the institution and authorized by the Veterinary Service of the Prefecture of Athens, as mandated by Greek legal requirements for animal experimentation (registration number 5522/24-10-2018). The unit is ISO 9001:2015 accredited and registered as a ‘user’ establishment according to Greek Presidential Decree 56/2013, in harmonization European Directive 2010/63 on the Protection of Animals Used for Scientific Purposes. Efforts were made to minimize the number of animals used and their suffering. The surgical procedures, post-operative procedures, daily care and termination procedures were supervised by veterinarians. For this study, we used five farm pigs (Landrace×Large White female pigs), with a body weight of 20–25 kg and aged 2.5–3 months at the time of the surgeries. In addition, we used three female Aachen minipigs of 20 kg aged ∼8 months old (Carfil, Oud-Turnhout, Belgium). This is a well-established swine breed in research (Pawlowsky et al., 2017; Schulze-Tanzil et al., 2020). All animals underwent an acclimatization period of 5 days minimum in the animal facility. Of note, the procedure described in this article is also based on the experience gained during the generation of acute SCIs in 20 farm pigs (Züchner et al., 2019).

### Generation of a controlled traumatic SCI

#### Sedation anaesthesia and identification of the surgical level

**Fig. 7.**
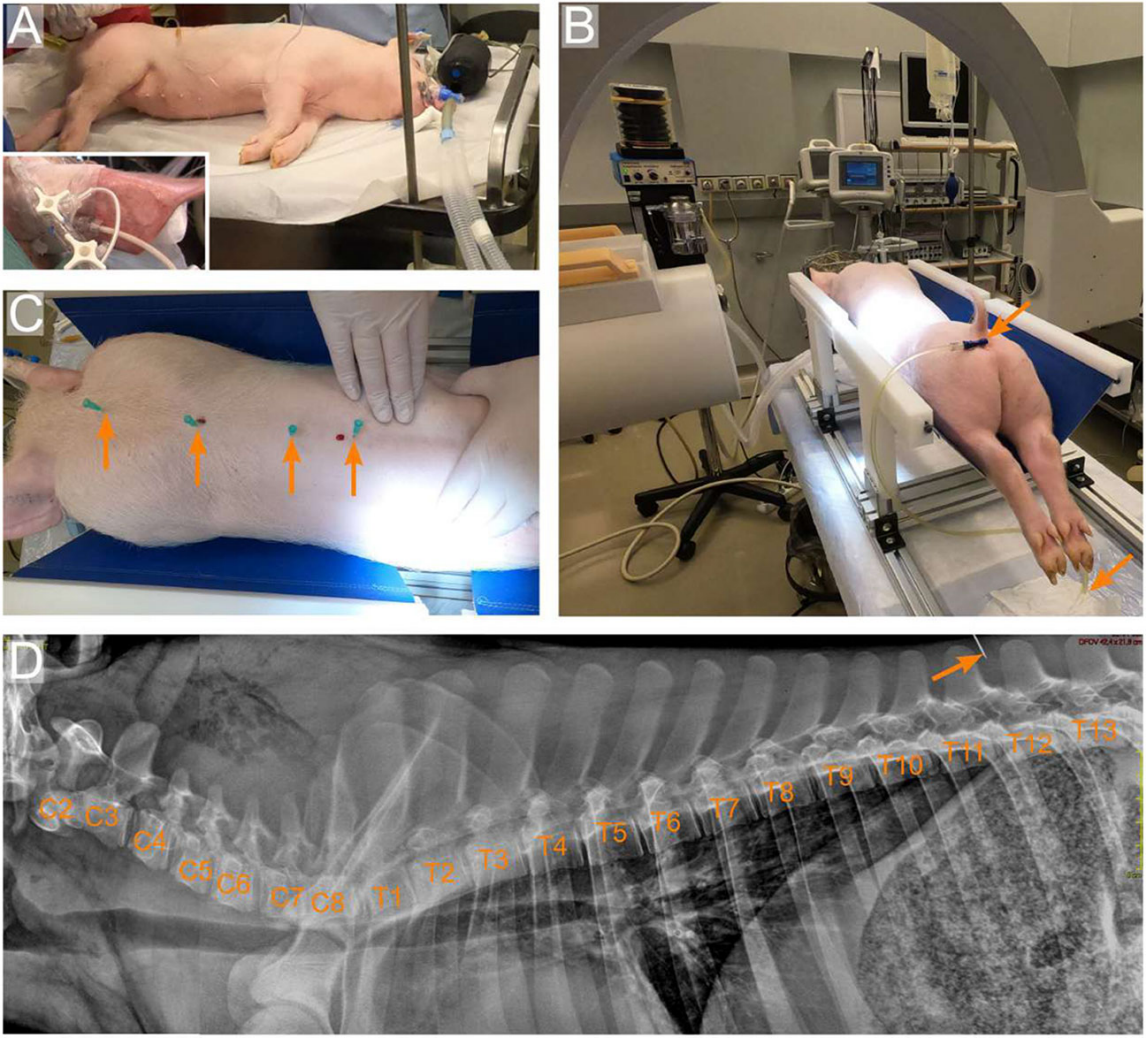
**Sedation and pre-operative X-rays.** (A) A 20 kg female pig sedated and intubated. The inset shows a catheter inserted and secured in an ear vein. (B) The pig is positioned on a pig operating table made of aluminium at the bottom and PEEK at the top. This allows the use of a C-arm. A trans-urethral catheter is inserted and further connected to a urine waste bag. (C) Insertion of needles (arrows), as landmarks, in the paravertebral musculature. (D) Sagittal X-ray showing the pig vertebral column from C2 to T13. Note the needle inserted between T12 and T13.

Food was withheld from pigs 12 h prior to the induction of anaesthesia, and body weight was measured before surgery to optimize the drug dosage. Each animal was pre-medicated with an intra-muscular (i.m.) injection of 10 mg/kg ketamine (100 mg/ml Ketamidor, Richter Pharma), 0.04 mg/kg atropine (1 mg/ml Atropine, Demo) and 0.4 mg/kg midazolam (50 mg/ml Dormicum, Roche). An auricular vein catheter was placed and secured with tape (Fig. 7A, inset). Anaesthesia was induced with an intra-venous (i.v.) injection of 0.9 mg/kg propofol (Propofol MCT/LCT/Fresenius 1%, Fresenius Kabi), and animals were intubated and attached to a veterinary anaesthesia machine (MDS Matrix, Model 2000, USA). Meloxicam (4 mg/kg i.m.) (5 mg/ml Melovem, Dopharma) and 7–10 mg/h (i.v.) fentanyl (0.5 mg/10 ml Fentanyl, Janssen) was administered before and during surgery, respectively, in order to achieve the maximum possible analgesia. Anaesthesia was maintained by a closed-circuit system, with inhalation of a mixture of 2.5% isoflurane (IsoVet, Piramal Healthcare) and oxygen (1.5 l/min) at a rate of 12–15 breaths/min. An anaesthesia-monitoring record was recorded every 15 min for the duration of the procedure and for 1 h post-surgery, including heart rate, respiration and arterial blood pressure, initially with a non-invasive technique. Basic vital signs were monitored during surgery using a Passport 2 (Datascope Corp., USA) monitor.

The pig was further moved onto the pig operating table (for construction details see Züchner et al., 2019). Next, a two-way, 8F (2.7 mm) silicon transurethral catheter was inserted and connected to a collecting bag (Fig. 7B), as described elsewhere (Ettrup et al., 2011). This can either be done at the start or at the end of the surgical procedure, as long as the pig is in deep sedation, to take advantage of the relaxation of the external urethral sphincter. As the injury strength does not necessarily correlate with loss of bladder function, we recommend using a bladder catheter, even for light injuries. The catheter remained in place for a week.

Intra-operative X-ray was performed with a C-arm (Philips BV Libra). To avoid image disturbances, we constructed the upper parts of the pig operating table in the plastic polyether ether ketone (PEEK). With palpation, we identified the last rib and further identified different levels of interest by placing needles in the paravertebral musculature and by taking successive X-rays (Fig. 7C,D). With a permanent marker, the skin was labelled to indicate the position of the vertebral levels of interest (T10–T12) and the surgical area.

#### Surgical tools and preparation of the surgical area

Prior to the surgery, the surgical tools were cleaned in a dishwasher at 50–60°C, placed in a metallic tray (Fig. S3A) and autoclaved in a metallic box. However, several parts of the impactor do not tolerate autoclaving, especially the load button sensor and laser displacement sensor. The impactor can be cleaned with alcohol, chlorhexidine or hydrogen peroxide; the impactor tip (PEEK) and nut cylinder (aluminium) can be autoclaved. This is especially important because they are close to or touching the surgical wound. Next, one or two tables were covered with sterile surgical drapes, following standard practice to preserve sterility, and the surgical tools were positioned regrouped by themes (Fig. S3B). The basic set of surgical tools required for this surgery is shown in Fig. S3C. The surgical area was further shaved and cleaned three times with cotton and chlorhexidine (Fig. S3D). A self-adhesive surgical drape was placed around the surgical area (Fig. S3E,F), and a tool bag was glued on the surgical drape. This is convenient to place tools frequently used by the surgeon, such as the monopolar cautery knife or suction.

#### Laminectomy

**Fig. 8.**
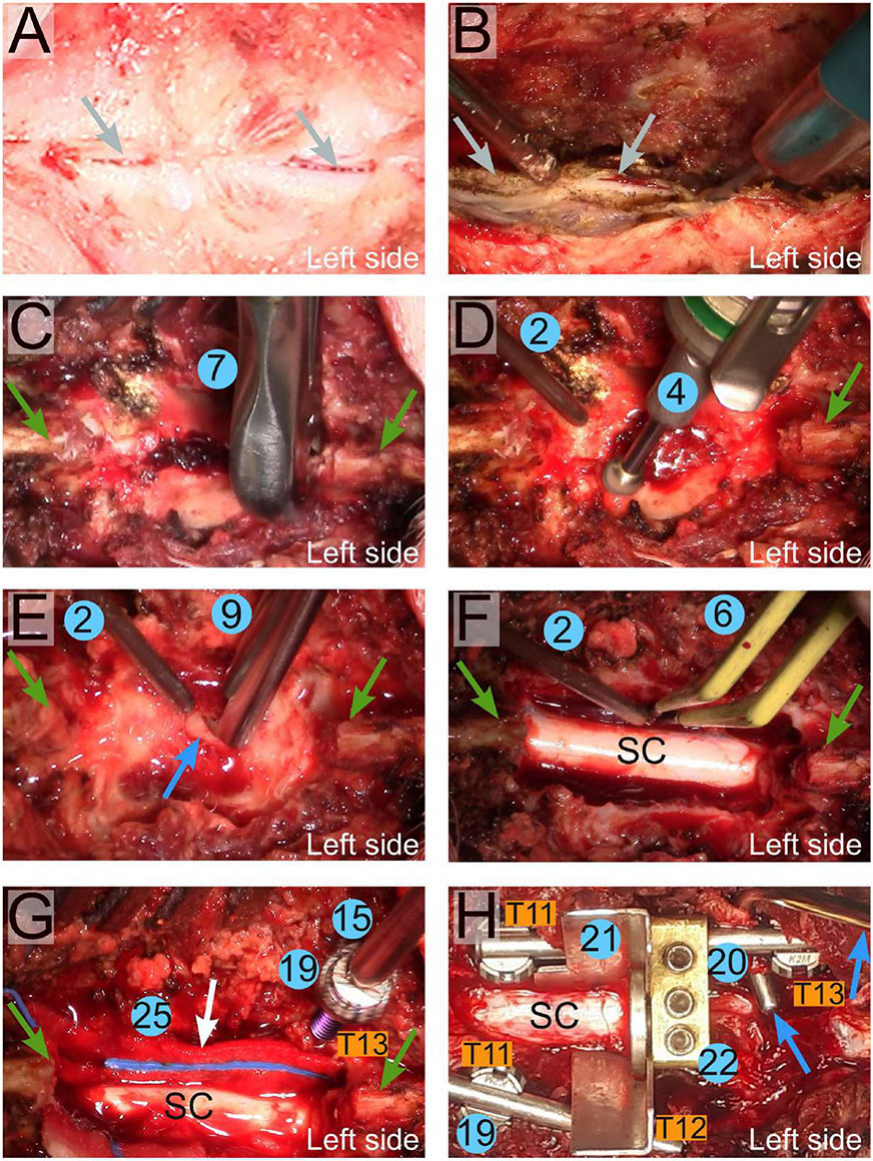
**Key steps for the generation of a controlled spinal cord injury.** (A) Identification of the spinal processes (grey arrows). (B) Mobilization of paravertebral muscles with a monopolar knife. (C) Removal of two spinal processes with a Leksell bone rongeur. The green arrows indicate the boundaries of the previous and the next spinal process. (D) Flattening of the laminae with a drill. (E) Laminectomy performed with a Love-Kerrison rongeur. The blue arrow points at the first opening. (F) A 2.5-level laminectomy is completed. Bleedings are controlled with bipolar electrocautery. (G) Insertion of a pedicle screw in the T13 right pedicle. The white arrow shows a neurosurgical sponge used to control bleeding. (H) The spinal cord support system is fully assembled. Pedicle screws are inserted in the left and right T11 pedicles and the left T12 and right T13 pedicles. A link element mounted on the right titanium bar binds the metallic plates that surround the spinal cord. The blue arrows show the hook of the vertebral suspension system; see also Züchner et al. (2019). Numbers indicate the elements shown in Fig. S3C. SC, spinal cord.

We performed an 8 cm posterior midline skin incision between T8 and T12 with a scalpel (Fig. S3C, element 1). With retractors (Fig. S3C, element 3) positioned at the cranial and caudal end of the incision, we enlarged the surgical area and identified the midline and spinous processes (Fig. 8A). We further incised the paravertebral muscles longitudinally on each side of the midline using the scalpel and mobilized the muscles with a monopolar cautery knife set on 20 W (Fig. 8B). When the lamina was reached, we removed the spinous processes of T11 and T12 with a Leksell bone rongeur (Fig. S3C, element 7; Fig. 8C). We further flattened and reduced the thickness of the lamina with a drill (Fig. S3C, element 4; Fig. 8D). To avoid overheating from the drill bit, it is necessary to flush it with 0.9% NaCl. We next identified the junction between the two laminae with a Murphy probe (Fig. S3C, element 8), and we introduced the tip of a Love-Kerrison rongeur into this space (Fig. S3C, element 9) to remove fragments of the laminae (Fig. 8E), resulting in a 2.5 segment laminectomy (Fig. 8F). At regular intervals during this process, we flushed the wound with 0.9% NaCl, and we controlled bleeding with bipolar electrocautery (Fig. S3C, element 6; Fig. 8F). Pronounced bleeding may occur by exposing dilated epidural veins upon removal of the medial part of the facet joints. To control this, we used TachoSil fibrin sealant patch (Baxter) soaked with 0.9% NaCl and covered by a neurosurgical sponge (Neurosorb, Vostra GmbH, Aachen, Germany; see also Fig. 8G, white arrow).

#### Fusion of vertebrae

We identified the pedicles tactile with a curved Murphy probe (Fig. S3C, element 8). The pedicles were further punctured with a pedicle probe (Fig. S3C, element 12), and we identified the inner part of the vertebral body with a pedicle feeler (Fig. S3C, element 13). In the minipigs we used, the bone is substantially more calcified compared to that of farm pigs of the same weight, and, to puncture the pedicle, it is necessary to use an electrical 2.5 mm drill. Next, we made a thread through the pedicle into the vertebra body using a 3.5 mm diameter tap (Fig. S3C, element 14). We further introduced a polyaxial screw (3.5×22 mm) (Fig. S3C, element 19) using a size 10 tapered screwdriver (Fig. S3C, element 15; Fig. 8G). This was repeated until four screws were placed in the right T11 and T13 and left T11 and T12 pedicles. It is important that the screws are not positioned too medially, as it could prevent the assembly of the spinal cord support system. Next, we cut two 3.5 mm diameter titanium rods (Mesa mini pedicle system) (Fig. S3C, element 20) with a rod cutter (K2M instruments 1101-90024) to fit the distance between the screws on each side, and we further attached them using a pedicle screw locker (Fig. S3C, element 17). If re-positioning is needed, it is possible to release the titanium rod using the pedicle screw unlocker (Fig. S3C, element 16).

#### Assembly of the spinal cord support device

The link element (Fig. S3C, element 22; Fig. 8H) was attached to the longest titanium rod (right side) almost facing the left T12 screw. It is important not to place it more caudally, as the left T12 screw would block the placement of the left plate (Fig. S3C, element 21). Similarly, if the placement of the link element is more cranial, it will create a conflict between the plate and the T11 screws. The link element was tightened to the titanium rod using a 2 mm Allen wrench (Fig. S3C, element 23). If not already done, it is important to loosen the screws that tighten the disc of the link element with the same 2 mm Allen wrench. The bent plate was then slid under the left side of the spinal cord and pushed gently towards the right side until the tip of the bent part could be visualized. The plate was attached to the link element by sliding its border between the disc and the edge of the link element (Fig. 8H). We further positioned the straight plate on the right side of the spinal cord so that the two end borders of the plates met. The straight plate was attached to the link elements in the same way, and the screws were tightened with a 2 mm Allen wrench to firmly press the plates against the link element and lock them in place.

#### Straightening the vertebral column

The vertebral column was suspended on a stand attached to the pig operating table, as previously described (Züchner et al., 2019). The stand is made of metallic parts that can be autoclaved. Hence, it can be assembled before the start of the surgery or during the procedure, depending of what the surgeon prefers. The suspension rods terminate with a hock placed under the titanium bar (Fig. 8H, blue arrows). The suspension rod is further pulled upwards until the vertebral column is straight. Tightening the screws on the stand immobilizes the suspension rods.

#### Impacting the spinal cord

After checking that the sensors are online and ready for acquisition, we armed the impactor using the lock mechanism, and we attached the articulated arm to the pig operating table, as previously detailed (Züchner et al., 2019). To avoid uncontrolled movements triggered by the injury, 10 mg/10 kg atracurium besilate (10 mg/ml, Tracrium, GSK) was injected i.v. for muscle relaxation. The impactor tip was first positioned between the two plates, 10 mm above the spinal cord. For a more precise positioning, it is best to stop the ventilation before approaching the impactor tip so that it touches the dura. The real-time readout from the force sensor is used in this process, as it indicates the contact with the dura by an increase of the load. It is important to have good communication between the surgeon and the person monitoring the sensor. Once the desired position was reached, the load sensor displayed values of 0.1–0.3 N, indicating contact between the dura and the impactor tip. The articulated arm could then be locked in position. The surgeon released the impactor, and the time was monitored. If it is desired that the static compression lasts for more than 1 min, it is then necessary to resume the ventilation. Note, that when the respirator is on, it generates a rhythmical pressure on the spinal cord that can be recorded by the sensors. Hence, it is important to standardize the volume and the rhythm of the ventilator. In this study, the static compression varied depending on the injury strength desired. During prolonged compression, we used a volume of 200 ml and a frequency of 12-15 breaths/min as ventilator settings.

#### Closing the wound

At the end of the desired compression time, the impactor was removed, and the suspension system and spinal cord support system were disassembled. In the first three pigs, we left the pedicle screws and titanium rods on the right side in place and removed the implants on the left side. As discussed earlier, we noticed that it was preferable to leave all implants for optimal stabilization of the vertebral column. This was done for all subsequent pigs. The surgical wound was further abundantly rinsed with saline and the bleeding controlled with bipolar cautery. Before closing the surgical wound, we flushed it with 500 mg/ml vancomycin (1 g vancomycin hydrochloride, Premier ProRx). The wound was closed by suturing the muscles and the skin in layers.

### Post-operative care

Cephalosporin (25 mg/kg, Zinacef, GSK) was administered at the beginning and by the end of the procedure. Post-operatively, the basic vital signs were monitored for 1 h, which corresponds to the awakening time of the animal. The ventilation was then stopped, but the endotracheal tube was kept for at least 15 min in case spontaneous breathing did not resume. The pigs also received 0.4 mg/kg meloxicam (5 mg/ml Melovem, Dopharma) i.m. for analgesia and 2.5 mg/kg enrofloxacin (50 mg/ml Baytril, Bayern) for 5 consecutive days.

Once awake, the pig was placed back in a 2×2 m cage. The sides of the cage were made with round bars to allow for social interactions with the pigs of the neighbouring cage. Fever, skin sores, signs of infections, food and water intake, and urinary and bowel functions were monitored daily.

### Locomotor behaviour

Before the surgery and once weekly after surgery, pigs were taken to an open space and their movements were video recorded at 250 Hz with a GoPro7 camera. The floor of the open space was covered by a rubber foam mat. Pigs were encouraged to walk in the desired direction with food reward. Locomotor behaviour and performance was evaluated by a blinded team member using the MPWS (Santamaria et al., 2018). This is a ten-grade ordinal scale assessing the evolution of natural recovery, ranging from no hindlimb movements to weight bearing, stepping and ambulation. Once pigs were able to ambulate, other parameters, including total standing-up-time with weight bearing, number of falls and normal hoof placement (NHP), describing distal motor control, were also used in order to completely and accurately describe end point-deficit profiles of each pig. The uptime criterion was normalized to 100%; NHP and number of falls were normalized to the total number of steps performed during the stand-up time and expressed in percentages. Whenever weight bearing and stepping was achieved, kinematic analyses were performed using deep learning software (DeepLabCut; Nath et al., 2019) to label anatomical joint landmarks [i.e. the iliac crest (IC), hip (H), knee (K), ankle (A), metacarpal (MTP) and hoof (Hf)]. Kinovea software (https://www.kinovea.org/) was further used to reposition any misplacement of the anatomical joint landmarks during video tracking sequences and to extract *x*, *y* coordinates. These were used to generate stick diagrams describing the motion of a single step, as well as angular excursion values, to account for differences between the pre- and post-operative conditions.

### Termination and spinal cord dissection

All pigs were terminated 7 weeks after SCI. The pigs were sedated and anaesthetized as described above. New X-rays were taken to evaluate the position of the implant (Fig. S1). The surgical area was defined by the scar still visible on the skin. We used the same approach as described above for skin opening, mobilization of muscles and to control bleeding. When approaching the laminectomy, we observed the presence of thick scar tissue (Fig. S2A) that took the place of the lamina and spinous processes. We removed it with a Leksell bone rongeur. In addition, there was epidural scar tissue attached to the dura (Fig. S2B). We carefully removed the epidural scar tissue with forceps and a dissector until the dura was free (Fig. S2C,D). To avoid uncontrolled movements, 10 mg/10 kg atracurium besilate (10 mg/ml, Tracrium, GSK) was injected i.v. for muscle relaxation. For SCI-1 and SCI-2, we opened the dura (Fig. S2E), cut the roots on either side (Fig. S2F) and removed the spinal cord. However, we found that an easier approach was to keep the dura intact, cut the roots and perform two extradural transections, rostral and caudal to the lesion. After rinsing in saline, the dura was removed in a dish using scissors and forceps. In all cases, the spinal cords were briefly rinsed in phosphate buffered saline (PBS) and immersion fixed in 4% paraformaldehyde diluted in PBS. All pigs were then euthanized with a 10 ml bolus dose (i.v.) of sodium pentobarbital (400 mg/ml Exagon, Richter Pharma).

### Post-mortem tissue processing and analysis

High-resolution 9.4T MRI T1 images were obtained as previously described (Züchner et al., 2019). Volume analyses was performed with Free-D software (http://free-d.versailles.inra.fr/; Andrey and Maurin, 2005).

For tissue sectioning, a mitre box dimensioned to pig spinal cord (40 mm length and 8 mm inner width) was built using plastic at the local workshop. Along the entire length of the box, multiple slots of 0.5 mm width were cut open at a 90° angle. The inter-slot distance was 2 mm. This allowed cutting slabs of the spinal cord of desired thickness, in this case 4 mm, using a razor blade guided through the slots. The slabs of spinal cord were further cryo-protected with 30% sucrose dissolved in PBS and embedded together in 7.5% gelatine from bovine bones (VWR) dissolved in a 15% sucrose–PBS solution (Nagamoto-Combs et al., 2016), together with an additional slab from an intact mouse spinal cord as a positive control for the downstream immunofluorescence. After immersion fixation in 4% paraformaldehyde diluted in PBS for 24 h, this assembly was frozen on dry ice and sectioned with a cryostat at 30 µm per section. The sectioned assembly was collected on positively charged microscopic slides (Superfrost Plus, Thermo Fisher Scientific).

Immunolabelling was done as previously described (Boulland et al., 2013). Briefly, the sections were washed in PBS and incubated in blocking solution [10% foetal calf serum (FCS) in Tris-base buffer with 0.5% Triton X-100 (TBST)] for 1 h. The primary antibodies were diluted in 1% FCS in TBST. Polyclonal rabbit anti-GFAP, (Agilent Dako, Z0334) was used at a 1:200 dilution together with chicken anti-NFH (Biosite, PLK592P) at a 1:3000 dilution. Polyclonal rabbit anti-NeuN (Abcam, ab177487) was used at a 1:500 dilution. The sections were incubated with primary antibodies overnight at room temperature. The tissue was further rinsed with 1% FCS in TBST, and secondary antibodies [Alexa Fluor 488 goat anti-rabbit (Invitrogen) alone or together with Alexa Fluor 647 goat anti-chicken (Invitrogen)] diluted at 1:500 were applied to the sections and incubated for 1 h at room temperature. The sections were further rinsed with PBS, incubated with Hoechst diluted in PBS (1:10,000), further rinsed with PBS and mounted with glycerol, PBS (1v:1v). For negative controls, the primary antibodies were not included in the primary antibody solution. Fluorescence images were obtained with a Zeiss 700 laser scanning microscope using identical scanning parameters for all sections. We used stitching scans to obtain survey images of the spinal cords.

## Supplementary Material

Supplementary information
