# Supplementary material for: How to generate graded spinal cord injuries in swine – tools and procedures
Source: Dis Model Mech. 2021 Aug 31;14(8):dmm049053. doi: 10.1242/dmm.049053 (PMC8419714; doi:10.1242/dmm.049053)
Supplement: Supplementary information [file dmm-14-049053-s1.pdf]

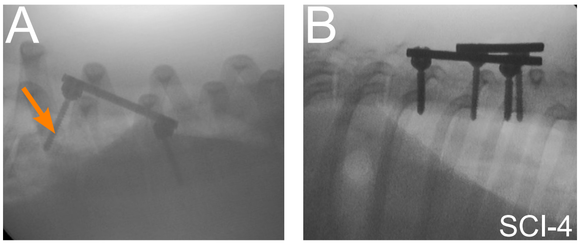

Fig. S1. Stabilisation of the vertebral column. (A) Insufficient stabilisation with unilateral implants revealed by intraoperative X-rays for SCI-3. (B) Bilateral implants for spinal column stabilisation done for all the remaining farm pigs and minipigs.

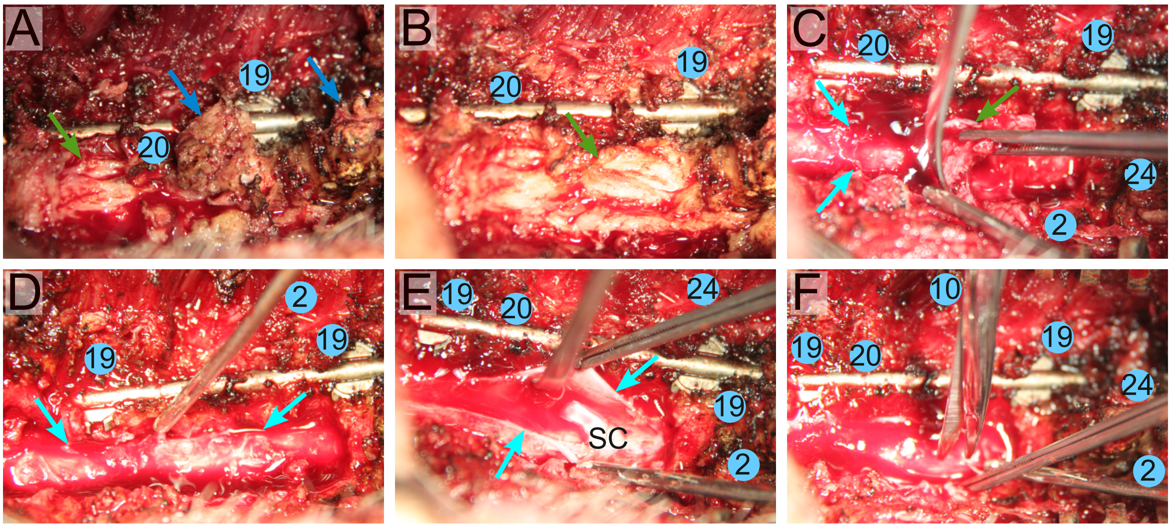

Fig. S2. Key steps for the removal of the spinal cord several weeks after spinal cord injury. (A) Removal of the thick scar-like tissue that replaced the lamina. (B,C) Removal of extradural scar tissue. (D,E) Opening of the dura. (F) Sectioning of the rootlets and transection of the spinal cord.

Disease Models & Mechanisms • Supplementary information

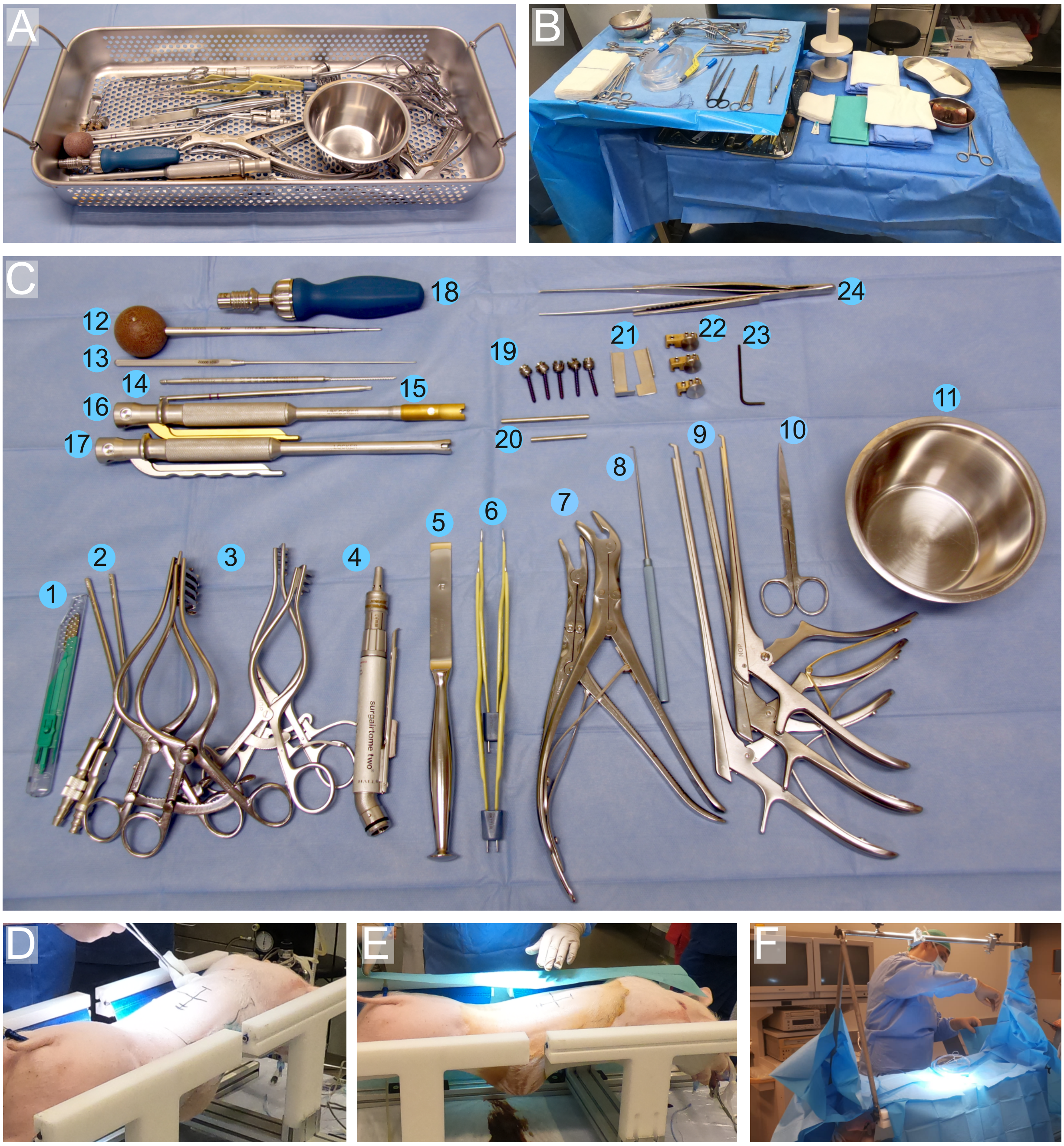
A
B
C
1
2
3
4
5
6
7
8
9
10
11
12
13
14
15
16
17
18
19
20
21
22
23
24
D
E
F

Fig. S3. Preparing for surgery. (A) Tools in an autoclaving tray. (B) Surgical tables where the tools are placed. (C) The minimum set of tools necessary to perform the surgical procedure.

1. Scalpel: Skin opening
2. Suction: Removal of blood and holding tissue
3. Retractor: Enlarging the surgical area
4. Drill: Trim bone
5. Large spatula: Mobilize muscles
6. Bipolar electrocautery: Surgical hemostasis
7. Leksell bone rongeur: Removal of large bone fragments
8. Murphy probe: Manipulate tissue
9. Love-Kerrison rongeur size 4-2: Removal of small bone fragments
10. Scissors
11. Small bucket: Contains saline solution to rinse the wound
12. Pedicle probe: Puncture the pedicle
13. Pedicle feeler: Identification of vertebral body after pedicle puncture
14. Tap, 3.5 mm: Drill through the pedicle. To be mounted on the handle (18)
15. Tapered screwdriver size 10: Insert polyaxial pedicle screws (19). To be mounted on the handle (18)
16. Pedicle screw unlocker: Release the titatium rod (20) from the head of the polyaxial pedicle screw (19)
17. Pedicle screw locker: Lock the titanium rod (20) into the head of the polyaxial pedicle screw (19)
18. Mini Axial AO Handle, Ratchet: Handle to fit elements 14 or 15
19. Mesa Mini Polyaxial screw (3,5x22 mm): Contribute to vertebral fusion. To be attached to the element 20
20. Titanium rod 3.5 mm diameter: Contribute to vertebral fusion. To be attached to the element 19
21. Spinal plates: To surround the lateral and ventral aspects of the spinal cord. To be attached to the element 22
22. Link element: To connect the spinal plates (21) with the titanium rods (20).
23. Allen wrench (2 mm): Lock the link element (22) with the titanium rod (20) and the spinal plates (21)
24. Anatomical and surgical forceps

(D) Cleaning of the surgical area. (E,F) Covering the non-surgical area with adhesive surgical blankets.

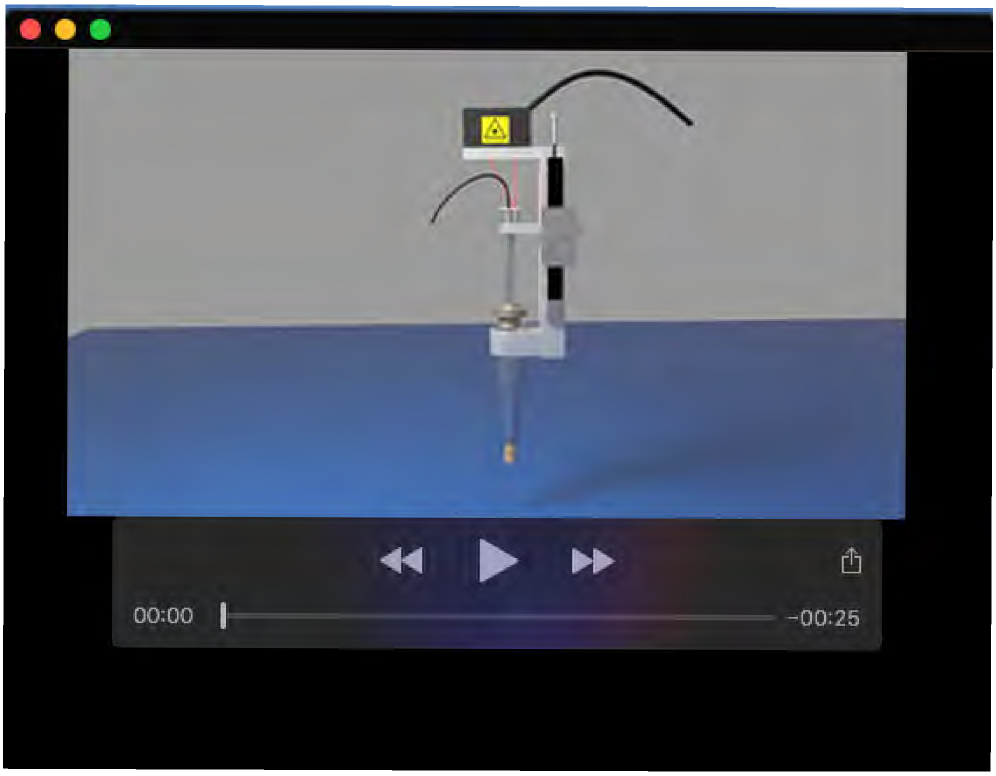

Movie 1. 3D model of the impactor.

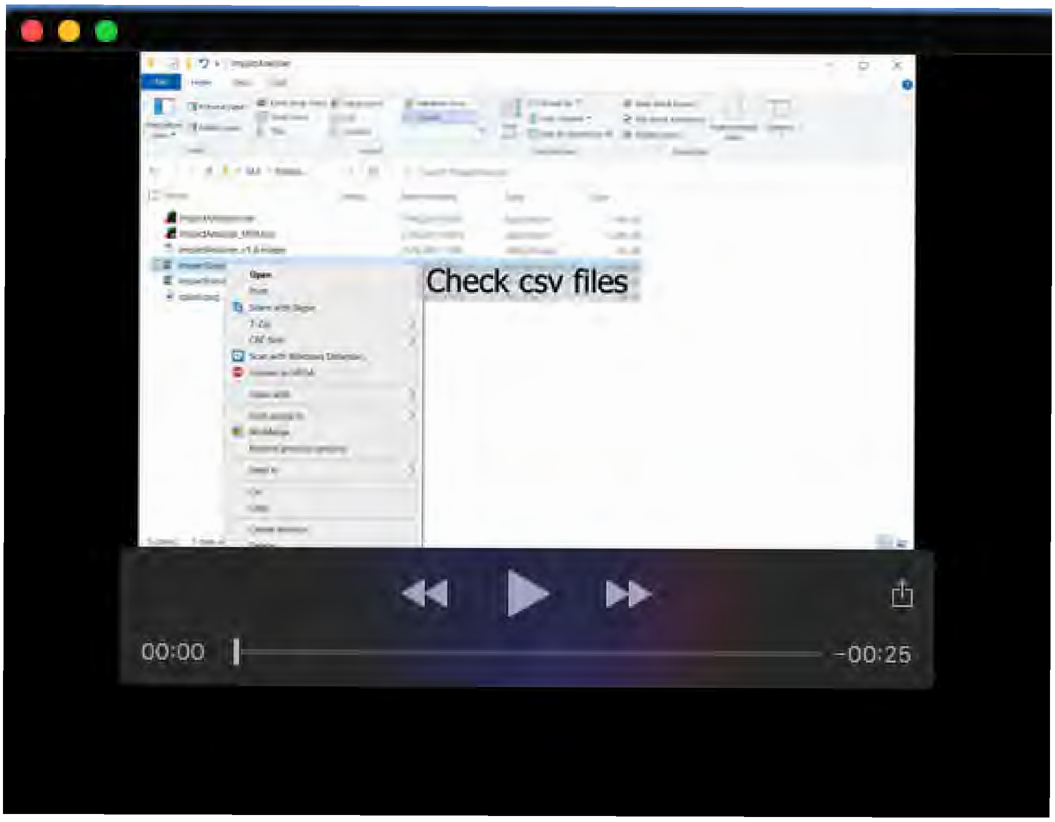

Movie 2. Tutorial video for using the MATLAB programme written to analyse the biomechanical characteristics of the impact based on raw data from sensors. Test data files are also provided.

Disease Models & Mechanisms • Supplementary information

Dataset 1. CAD file.

Click here to download Dataset 1

Dataset 2. MATLAB code.

Click here to download Dataset 2

Dataset 3. Demonstration files.

Click here to download Dataset 3

Dataset 4. User manual.

Click here to download Dataset 4
